# Construction of Phantoms for MR Electrical Properties Tomography (From Structure to Composition): A Guideline From the ISMRM Electro‐Magnetic Tissue Properties Study Group

**DOI:** 10.1002/jmri.70059

**Published:** 2025-08-20

**Authors:** Ilias I. Giannakopoulos, Alessandro Arduino, Cornelis A. T. van den Berg, Zhongzheng He, Kyu‐Jin Jung, Dong‐Hyun Kim, Riccardo Lattanzi, Jessica A. Martinez, Thierry Meerbothe, Freddy Odille, Adriano Troia, Luca Zilberti, Stefano Mandija

**Affiliations:** ^1^ Department of Radiology, Bernard and Irene Schwartz Center for Biomedical Imaging and Center for Advanced Imaging Innovation and Research (CAI^2^R) New York University Grossman School of Medicine New York New York USA; ^2^ Istituto Nazionale di Ricerca Metrologica Torino Italy; ^3^ Computational Imaging Group for MR Therapy and Diagnostic, Department of Radiotherapy University Medical Center Utrecht Utrecht the Netherlands; ^4^ IADI U1254, INSERM and Université de Lorraine Nancy France; ^5^ Department of Electrical and Electronic Engineering Yonsei University Seoul Republic of Korea; ^6^ National Institute of Standards and Technology (NIST) Boulder Colorado USA; ^7^ Department of Physics University of Colorado Boulder Boulder Colorado USA; ^8^ CIC‐IT 1433, Inserm Université de Lorraine and CHRU Nancy Nancy France

**Keywords:** electrical tissue properties, phantom fabrication, phantom guidelines

MR‐based Electrical Properties (EPs) Tomography (MR‐EPT) denotes non‐invasive electrical conductivity (*σ_e_*) and permittivity (*ε*_*r*_) mapping methods using MR measurements. The lack of standardized tissue‐mimicking phantoms hinders reproducibility studies and method comparisons. For example, the NIST/ISMRM MRI system phantom has contributed significantly to standardization efforts in relaxometry, underscoring the importance of having similar benchmarks for MR‐EPT. The guidelines presented herein outline the importance of MR‐EPT phantom design and construction, focusing on structure, composition, and reliability.

## 1 Phantom Structure

The structure should mimic the shape and size of the anatomy of interest. For example, the head can be modeled using a simple spherical geometry or with anatomically realistic designs such as the one presented in [1]. The abdomen can be represented using large elliptic cylindrical phantoms or specialized holders that conform to human anatomy and are commonly used for training purposes [2, 3]. This allows for testing compatibility with standard receive coils and allows the use of established methods to mitigate *B*_0_ inhomogeneity artifacts and spurious phase contributions. Phantoms that are too small to sufficiently load the coil could detune the coil elements or couple opposite receivers. Moreover, phantoms with translational symmetry along the longitudinal axis and significantly larger geometry in the Frankfort horizontal orientation (*B*_0_ direction) than the anterior‐to‐posterior and right‐to‐left (e.g., cylinders with length ≥ 4× radius) orientations are ideal for 2D MR‐EPT methods since the derivatives along *z* of *B*_1_ in the midplane of a birdcage coil are approximately null. However, they do not provide a good validity test for human anatomies.

**FIGURE 1:**
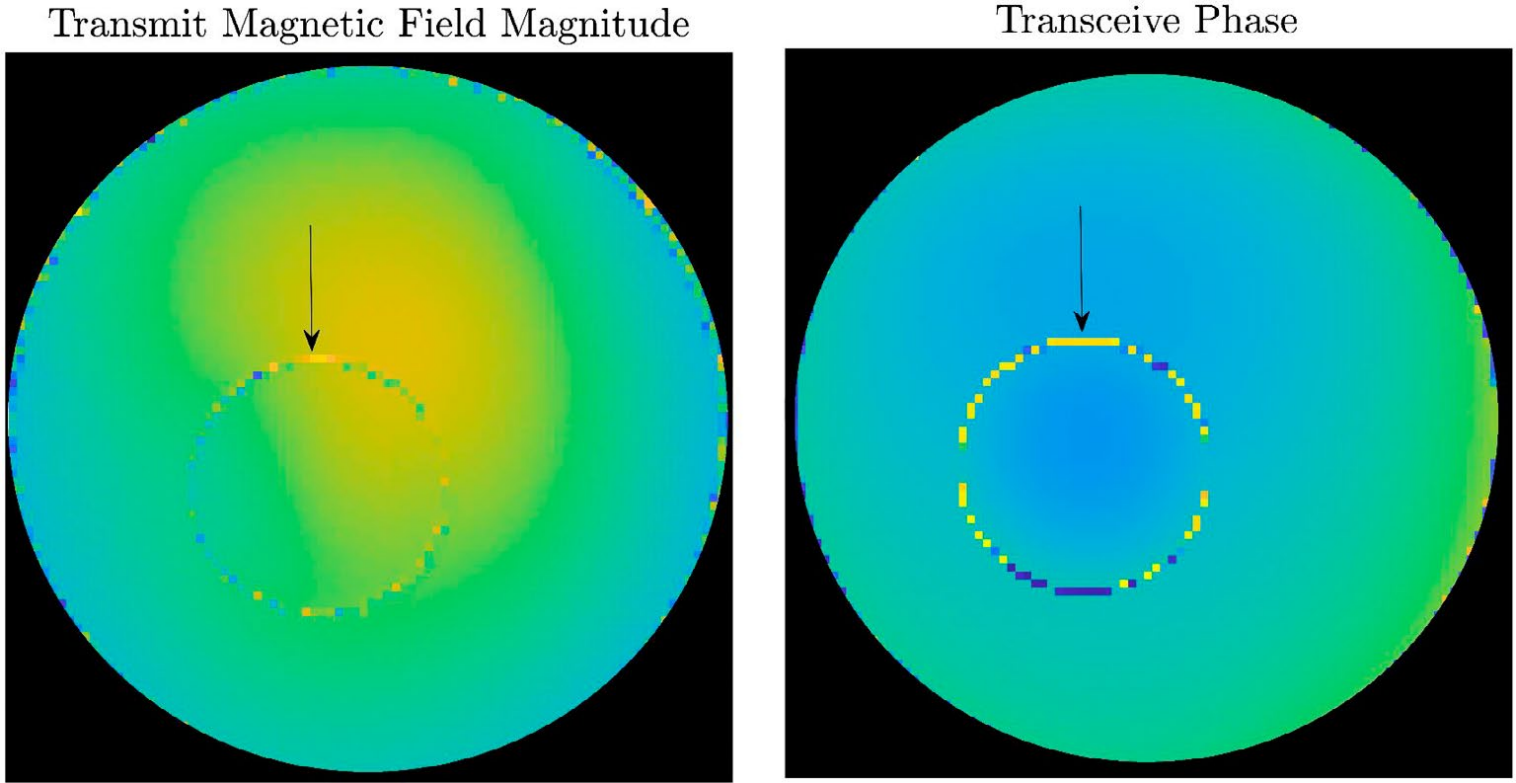
Magnitude of the transmit magnetic field (left) and transceive phase (right) for an axial slice of a two‐compartment cylindrical phantom (details on compartment EP values are provided in the next sections). The phantom was scanned at 2.5 mm isotropic resolution using the RF map sequence [8]. The plastic boundary separating the two compartments was approximately 2.5 mm thick. This boundary region is noisy due to the lack of MR signal in both the magnitude and phase maps.

To discriminate between different tissue EPs, heterogeneous phantoms should be used. The phantom's compartments can be separated with plastic boundaries (e.g., for liquid‐based compounds) or kept in direct contact (gel‐based compounds). Liquid‐based compounds may be suboptimal due to the risk of flow artifacts in images, which are avoided in gel‐based compounds. Gel‐based compounds may be put in direct contact [4], but electrolyte diffusion between compartments may alter the conductivity and internal geometry near the interfaces, as highlighted in [5, 6, 7]. To avoid these issues, solid structures can be used to separate different compartments. However, due to the significant mismatch in EP between the plastic dividers and adjacent tissue‐mimicking materials, these interfaces introduce localized artifacts [1]. In particular, at least one voxel in the magnitude transmit magnetic field is consistently corrupted by noise near the boundary. Figure 1 illustrates this boundary effect in a two‐compartment cylindrical phantom, demonstrating how these image artifacts around the plastic dividers limit EP mapping assessment in small structures. Precise quantification of EP reconstruction errors related to plastic boundary thickness and material composition has not yet been established in MR‐EPT literature.

## 2 Phantom Composition

Phantoms should use deionized water as the solvent. Sodium chloride (NaCl) can be added to raise conductivity [5, 9, 10, 11]. Permittivity can be reduced using low‐permittivity materials such as polyvinylpyrrolidone (PVP) and sucrose. Although more costly, PVP is preferred, as sucrose can lead to stability issues over time, degradation in highly concentrated solutions, caramelization when exposed to high temperatures, and *T*_2_* shortening that may lower the SNR [12]. Ethanol and other organic solvents can also be used for permittivity reduction, but they have either poor water solubility, are volatile, or require light‐proof containers [5]. Glycerol can also be used, but it can induce chemical shift artifacts [11] and its high viscosity limits its useful concentration to around 30% and 50% for gel‐based and liquid‐based phantoms, respectively. Barium titanate (BaTiO_3_) or calcium titanate (CaTiO_3_) can increase permittivity, and due to their low solubility, osmosis is negligible. BaTiO_3_ is often preferred since it requires a lower concentration to achieve the desired permittivity value [13]. Agar or gelatin can be utilized for phantoms that require low heat diffusivity (to assess RF coil safety). Preservatives such as CuSO_4_, Proclin, or benzoic acid can enhance stability over time. Toxic and reactive preservatives like sodium azide should be avoided.

Relaxation times, particularly *T*_1_, can be adjusted with paramagnetic salts such as gadolinium chloride (GdCl_3_), copper(II) sulfate (CuSO_4_), or manganese(II) chloride (MnCl_2_) [14]. GdCl_3_ requires a lower concentration as a *T*_1_‐shortening agent [14], which helps minimize diffusion effects in phantoms without plastic boundaries, but it is toxic and has a pronounced impact on EP due to its high ionic charge. Agar and gelatin can also be used as *T*_1_ and *T*_2_ shortening agents [1, 11, 14, 15].

Phantom components should be weighed before construction to minimize errors in concentrations and potential mass loss during the high‐temperature steps of the mixing process. Once the Larmor frequency and target EPs are established according to the organ or the tissue of interest (e.g., brain, prostate, liver, etc.), the required amounts of PVP and NaCl may be calculated using, for example, the software outlined in [12].

Realistic tissue EPs should be targeted in phantom preparations, for example, relative permittivity < 120, conductivity < 2.5 S/m for 3 T [1]. When the conductivity is negligible, high permittivity can lead to interfering *B*_1_ patterns due to its inverse relationship with the wavelength, especially at fields ≥ 3 T [9]. High conductivity may give rise to *B*_1_ inhomogeneities due to eddy currents [9], which at fields ≥ 3 T can impair the performance of EPT methods that are based on specific assumptions that may no longer hold (namely, the transceive phase assumption, or negligible *B*_*z*_ gradient).

## 3 Example Procedure for Phantom Preparation

First, weigh the appropriate amount of water in a sealable borosilicate glass bottle. Second, dissolve the NaCl in the water. Third, gradually add the PVP (if used) while stirring vigorously. If the PVP concentration exceeds 30%, heat the solution to 60°C in a microwave oven. Typically, 500 g of solution is heated for 4 min at 900 W in a commercial microwave oven. Depending on PVP concentration, this procedure could be repeated, keeping the solution under agitation until it becomes fully transparent with a slight amber tint [16]. After this stage, agar or gelatin can be added for gel‐based phantoms. The mixture should be heated up to allow them to fully dissolve (agar: > 95°C, gelatine: > 70°C). Finally, during the cooling step under agitation (at room temperature), benzoic acid (or other preservatives) and the paramagnetic salt (if used) must be added for long‐term stability to prevent organic degradation and to tune the phantom relaxation times, respectively. Pouring into the phantom scaffold should be done carefully when the solution temperature is > 65°C, since the higher viscosity traps air bubbles at lower temperatures. Gentle shaking of the hot solution helps raise the bubbles to the surface. Alternatively, one can vacuum the material [17] or keep it in a water bath for 45 min before allowing it to cool to room temperature [18]. For gel‐based heterogeneous phantoms, solidification of the first gel is required before pouring the next one [4]. The application of this procedure with the quantities reported in Table 1 would result in two materials (C‐1 and C‐2) mimicking the electromagnetic behavior of cerebrospinal fluid and gray matter when exposed to the RF field of a 3 T MRI scanner.

**TABLE 1:** Example recipe for a two‐compartment (C‐1, C‐2) phantom used for EPT reconstruction at 3 T MRI.

	Recipe			Targets		Measurements			
	PVP	NaCl	MnCl_2_	*ε*_*r*_	*σ*_*e*_	*ε*_*r*_	*σ*_*e*_	*T*_1_	*T*_2_
Units	g	g	mg	—	S/m	—	S/m	ms	ms
C‐1	131.19	18.32	3.5	73.6	2.17	74.9 ± 3.8	1.97 ± 0.10	1080 ± 203	242 ± 12
C‐2	433.96	9.41	5.3	60.9	0.60	63.8 ± 3.2	0.63 ± 0.03	875 ± 148	423 ± 8

*Note*: The recipe is normalized to 1 kg of water. The relative permittivity (*ε*_*r*_) and the conductivity (*σ*_*e*_) were measured with a dielectric probe (Agilent, Santa Clara, CA), at 22.1°C and 128 MHz. The phantom's holder was constructed using cast acrylic. *T*_1_ and *T*_2_ values were measured with MP2RAGE and CPMG sequences, respectively.

## 4 Phantom Lifetime

A quality assurance protocol should be put in place to monitor the temporal stability of the phantom. However, since the criteria for determining when a phantom should be decommissioned are yet to be defined, reporting the lifetime of the phantom in publications can help understand the possible discrepancies between expected (from constructions) and measured EPs.

## 5 Reporting

**FIGURE 2:**
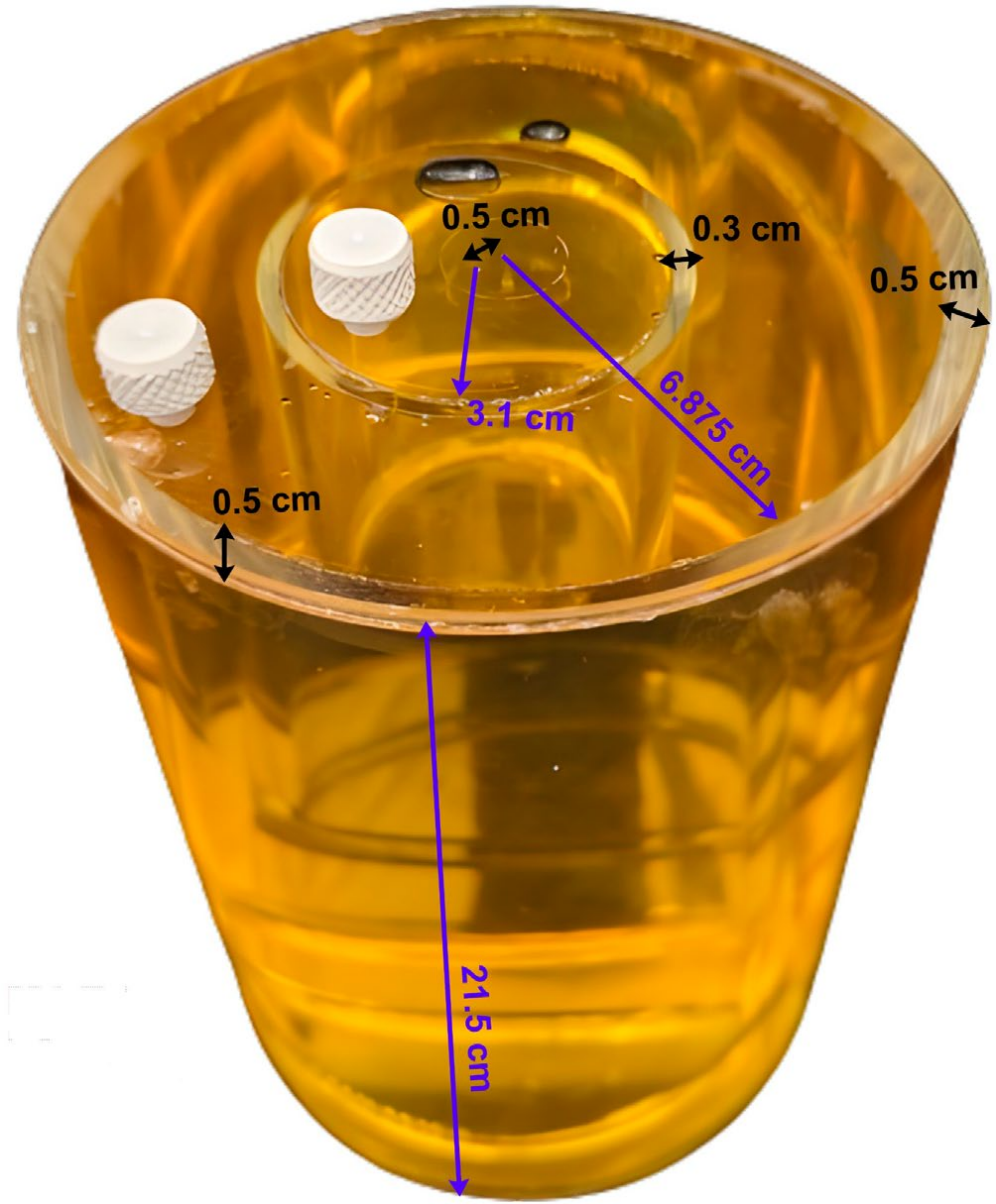
Schematic representation of a two‐compartment cylindrical liquid phantom used for EP mapping in [20]. The phantom consists of an outer hollow cylindrical and an inner cylindrical compartment. The total length of the phantom is 21.5 cm. The inner radius of the hollow cylinder is 6.875 cm, while the inner cylinder has an inner radius of 3.1 cm and is offset by 0.5 cm from the central axis of the hollow cylinder. The scaffold that contains the liquid solutions has a uniform thickness of 0.5 cm, except at the interface between the two compartments, where it is reduced to 0.3 cm.

When reporting phantom recipes, all concentrations should be expressed in terms of mass/mass (g/kg, mg/g), as it is independent of temperature fluctuations. Considering the variability of EPs with Larmor frequency and temperature, we recommend reporting the target EP values for a given frequency and temperature (e.g., as calculated using [12]), and when feasible, the measured EPs with an open‐ended coaxial probe [5, 19] (see an example in Table 1), including probe model. The sample characterized with the coaxial probe should be large enough to mitigate measurement errors. To improve the precision, we suggest repeating probe measurements five times and reporting the average and standard deviation values. If measurements last longer than 1 h, it is advisable to report the sample temperature before and after the experiment. If the *T*_1_ and *T*_2_ were modulated, it is also beneficial to report them. The type of plastic used for the phantom scaffold should also be reported. Finally, we recommend including a figure that shows the geometry and dimensions (compartment size and boundary thickness) to facilitate reproducibility, as in Figure 2.

## Disclosure

Certain commercial equipment, instruments, software, or materials are identified in this paper in order to specify the experimental procedure adequately. Such identification is not intended to imply recommendation or endorsement by NIST, nor is it intended to imply that the materials or equipment identified are necessarily the best available for the purpose.
